# Epidemiology of antimicrobial resistance in commercial eggs across different production systems in Spain

**DOI:** 10.1186/s13567-026-01798-8

**Published:** 2026-06-25

**Authors:** Ana Marco-Fuertes, Marta Cerdà-Cuéllar, Teresa Ayats, Marta Muñoz-Baquero, Clara Marin, Laura Montoro-Dasi

**Affiliations:** 1 Facultad de Veterinaria, Instituto de Ciencias Biomédicas, Universidad Cardenal Herrera-CEU, CEU Universities, Alfara del Patriarca, 46115 Valencia, Spain; 2 Unitat Mixta d’Investigació IRTA-UAB en Sanitat Animal, Centre de Recerca en Sanitat Animal (CReSA), Campus de la Universitat Autònoma de Barcelona (UAB), 08193 Bellaterra, Catalonia Spain; 3 Programa de Sanitat Animal, Centre de Recerca en Sanitat Animal (CReSA), IRTA, Campus de la Universitat Autònoma de Barcelona (UAB), 08193 Bellaterra, Catalonia Spain

**Keywords:** Antimicrobial resistance, commercial eggs, *Escherichia coli*, laying hens, production systems, public health

## Abstract

The European poultry sector is transitioning towards alternative production systems, driven by consumer concerns about animal welfare. However, these systems, by providing access to the outdoors, increase exposure to the environment and wildlife, raising the risk of disease spread and antimicrobial resistance (AMR). The objective of this study was to evaluate the occurrence of AMR and multidrug resistance (MDR) in *Escherichia coli* isolates from commercial eggs from the four production systems in Spain: organic, free-range, floor and cage, and their genetic diversity using pulsed-field gel electrophoresis (PFGE) analysis. To this end, 1200 eggs were collected from the main supermarket chains over the course of a year. The shell and contents were analysed, evaluating the susceptibility of isolates to 15 antimicrobials. A total of 49 *E. coli* isolates were found, only on the shell. Overall, resistance to at least one antimicrobial was found in 69.4% of the isolates, and 44.9% showed MDR. When compared, the prevalence of MDR was significantly higher in organic systems (61.5%) than in cage systems (22.2%; *p* < 0.05). Moreover, PFGE analysis revealed great genetic diversity with 37 distinct pulsotypes, observing that *E. coli* isolates with similar genetic profiles presented different AMR patterns. These results suggest that the outdoor environment and contact with wildlife are crucial for AMR acquisition, even in organic systems with restricted antimicrobial use. It is imperative to implement rigorous perimeter and environmental biosecurity measures in poultry production systems to mitigate the spread of AMR, addressing this public health challenge through a comprehensive One Health perspective.

**Supplementary information:**

The online version contains supplementary material available at 10.1186/s13567-026-01798-8.

## Introduction

Eggs are considered a basic dietary component worldwide owing to their exceptional nutritional content. They are regarded as a readily accessible source of high-quality protein, vitamins and minerals [1]. In recent years, there has been an increased focus on their potential health benefits, with research indicating the presence of bioactive compounds, both in egg yolk and albumen, that may contribute to overall health and wellbeing [2, 3]. Their gastronomic versatility and wide socio-cultural acceptance serve to consolidate them as a staple in many international cuisines, thereby driving an upward trend in their global consumption and production [3]. For all these reasons, it is one of the most important sectors worldwide.

Within the European Union, Spain is the third largest egg producer, accounting for 14.4% of the total, behind France and Germany [4]. This continuously expanding sector is undergoing a significant transition towards alternative farming systems, moving away from traditional cages in developed countries [5]. This transformation can be attributed primarily to mounting consumer concern for animal welfare. This trend is exemplified by the European Citizens' Initiative entitled *'End the Cage Age'*, which, with the backing of the European Commission, calls for a legislative transition towards more ethical and sustainable production models [6]. The proposal calls for the phased elimination of the enriched cage system (code 3), with a shift towards floor (code 2), free-range (code 1) and organic (code 0) production methods.

However, the shift to alternative cage-free systems, notably those providing outdoor access, gives rise to novel and intricate health and biosecurity concerns [7, 8]. As a result, one of the main concerns is the close contact poultry can have with wildlife animals cohabiting the same environment, which exposes them to multiple challenges. One of those, is the management of antimicrobial resistance (AMR), a global public health problem with serious economic repercussions for the livestock sector, not only affecting animals, but also the food chain, compromising the safety of the final product [9]. The transmission of resistance genes between bacteria – whether pathogenic or non-pathogenic – is favoured in environments where environmental control is limited [10]. Whilst cleaning and disinfection protocols are more easily managed in cage or floor (indoor) systems, in free-range and organic systems, the continuous exposure of animals to the outdoor environment and wildlife significantly reduces the effectiveness of these measures [11]. This scenario poses a significant challenge to biosecurity and AMR control in contemporary poultry farming. In this context, the commensal bacterium *Escherichia coli* has been extensively documented and employed as a sentinel bacterium in AMR epidemiology studies. This is primarily owing to its global distribution and its capacity to acquire and harbour AMR genes, thereby facilitating the assessment of AMR genes acquired through the environment. Thus, the aim of this study was to assess the relationship and AMR occurrence in the sentinel bacteria *E. coli* isolated from commercial eggs in different layer production systems in Spain.

## Materials and methods

### Experimental design and sample collection

From October 2023 to June 2024, a total of 1200 eggs from the four different systems (0: organic, 1: free-range, 2: floor, 3: cage) were collected. A total of 100 pools of 12 eggs each were processed for the recovery of *E. coli*, resulting in 25 pools per production system. Its traceability was monitored using the Spanish REGA code (from its Spanish acronym *Registro General de Explotaciones Ganaderas*) for each farm.

To this end, eggs were collected from the six primary largest retail distributors in Spain, which account for the majority of the Spanish market share. In each store, the purchasing of eggs was adjusted to reflect the available supply of each production system.

### *Escherichia coli* isolation

For the isolation of *E. coli*, all the eggs in each dozen purchased were pooled, and each pool was considered as one sample. The surface and the content of the eggs were evaluated separately. On one hand, in order to isolate *E. coli* present in the surface of the eggshell, all the eggs of the dozen were placed in a sterile zip bag and were washed for a duration of 2 mins in 9 mL of buffered peptone water (BPW, Scharlau^®^, Barcelona, Spain); this was considered to be a 1:10 v/v ratio of the eggshells. Next, in order to assess the presence of *E. coli* in the egg contents, once all the eggs were washed, their surfaces were disinfected with 70% alcohol and left to dry. The eggs were then cracked into a new sterile zip bag. The contents of the 12 eggs were weighed, and BPW was added at nine times the sample weight to obtain a 1:10 dilution (1:10 w/v). Then, all the zip bags with the BPW eggshell wash and egg contents were incubated at 37 ± 1 °C for 24 ± 2 h. Subsequently, each pool was inoculated on Tryptone Bile X-glucuronide agar (TBX; Scharlau^®^, Barcelona, Spain) and incubated at 37 ± 1 °C for 24/48 h. One blue colony compatible with *E. coli* was selected and streaked onto nutrient agar plates (Scharlau^®^, Barcelona, Spain) and incubated at 37 ± 1 °C for 24 ± 2 h to obtain pure cultures. The isolates were confirmed as *E. coli* by biochemical tests using the API 20E test (BioMerieux, Marcy l’Etoile, France). All the confirmed isolates were stored at −80 °C for further studies, each preserved in 1 mL of 20% glycerol for cryopreservation.

### Antimicrobial susceptibility testing

All the confirmed *E. coli* isolates were tested for antimicrobial susceptibility using a minimum inhibitory concentration (MIC)-based broth microdilution method with the EUVSEC3 Sensititre plates (Thermo Scientific, Madrid, Spain), which includes the antimicrobials set out in Decision (EU) 2020/1729. A total of 15 antimicrobials from 12 different classes were tested: two aminoglycosides (amikacin; AMI 2–32 μg/mL and gentamicin; GEN 0.5–8 μg/mL), two quinolones (ciprofloxacin; CIP 0.12–1 μg/mL and nalidixic acid; NAL 0.5–16 μg/mL), two cephalosporins (cefotaxime; CTA 0.25–4 μg/mL and ceftazidime; CTZ 0.25–8 μg/mL), one penicillin (ampicillin; AMP 2–16 μg/mL), one tetracycline (tetracycline, TET 2–32 μg/mL), two folate inhibitory pathways (sulfamethoxazole; SME 8–512 μg/mL and trimethoprim; TRI 0.25–16 μg/mL), one carbapenem (meropenem; MER 0.12–2 μg/mL), one glycylglycine (tigecycline; TIG 0.5–4 μg/mL), one polymyxin (colistin; COL 1–16 μg/mL, one amphenicol (chloramphenicol; CHL 8–64 μg/mL) and one macrolide (azithromycin; AZI 2–64 μg/mL).

The analyses were conducted according to the manufacturer’s instructions, as previously described [12]. The MIC values were interpreted as resistant (R), intermediate (I) or susceptible (S), according to the clinical breakpoints established in Decision (EU) 2020/1729 and the 2026 EUCAST guidelines [13]. The isolates were considered multidrug resistant (MDR) when they showed resistance to three or more classes of antimicrobials [14].

### Molecular typing of *E. coli* isolates

Genotyping of *E. coli* isolates was performed by pulsed-field gel electrophoresis (PFGE) according to the PulseNet standardised protocol. The genomic DNA of the isolates was digested with XbaI restriction enzyme (Roche Applied Science, Indianapolis, IN, USA). The resulting PFGE band patterns were analysed using Fingerprinting II v3.0 software (Bio-Rad, Hercules, CA, USA). Similarity matrices were calculated using the Dice coefficient with a band position tolerance of 1.0%, and cluster analysis was performed by the unweighted-pair group method with arithmetic mean (UPGMA). A cut-off of 90% was used for the determination of the different profiles (PFGE type or pulsotype).

### Statistical analysis

A generalised linear model (GLM) with a probit binary link function was fitted to the data in order to investigate whether there was an association between the categorical variables (production system, season and supermarket chain) and the appearance of AMR and MDR. In addition, a GLM with a probit binary link function was also used to evaluate whether there were differences between the antimicrobials studied. A *p* value < 0.05 was considered to indicate a significant statistical difference. The data are presented as least squares means ± standard error of the least squares means. The analyses were carried out using a commercially available software application (SPSS 29.0 software package; SPSS Inc., Chicago, IL, USA).

## Results

### Samples collected

In total, the 1200 eggs analysed in 100 pools (25 per production system) were from 34 farms located in 15 out of 50 regions of the Spanish Peninsula: A Coruña (*n *= 3), Cuenca (*n* = 4), Guadalajara (*n* = 1), Lugo (*n* = 2), Madrid (*n* = 1), Murcia (*n* = 1), Pontevedra (*n* = 1), Segovia (*n* = 1), Sevilla (*n * = 1), Tarragona (*n* = 1), Teruel (*n* = 1), Toledo (*n* = 4), Valencia (*n* = 7), Valladolid (*n* = 1) and Zaragoza (*n* = 5).

### *E. coli* prevalence

*E. coli* prevalence in the eggshells was 49% (49/100) while no *E. coli* was recovered from the eggs’ content. Of the total of 49 *E. coli* isolates, 13/49 (26.5%), 14/49 (28.6%), 13/49 (26.5%) and 9/49 (18.4%) were from organic, free-range, floor and cage production systems, respectively. Nevertheless, there were no significant statistical differences in the prevalence of *E. coli* among the different production systems, season of the year or large-scale retailers. Additional file 1 shows all the information regarding the farms sampled per region and their ID, and the number of isolates per farm and their ID.

### Antimicrobial susceptibility

**Figure 1:**
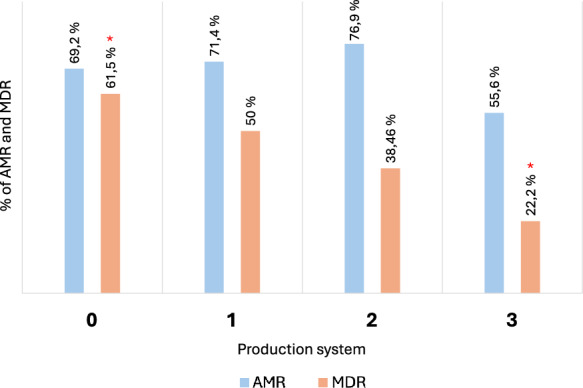
**Percentage of antimicrobial resistance and multidrug resistance depending on the production system**. AMR: antimicrobial resistance, MDR: multidrug resistance, 0: organic production system, 1: free-range production system, 2: floor production system, 3: cage production system, *: the superscript indicates statistically significant differences (*p* value < 0.05).

Resistance to at least one antimicrobial was revealed by the broth microdilution method in 69.4% (34/49) of the *E. coli* isolates and 44.9% (22/49) were MDR. Statistically significant differences were observed between cage (code 3) and organic (code 0) production systems, with a higher proportion of MDR isolates in organic farms (*p* value < 0.05) (Figure 1).

**Figure 2:**
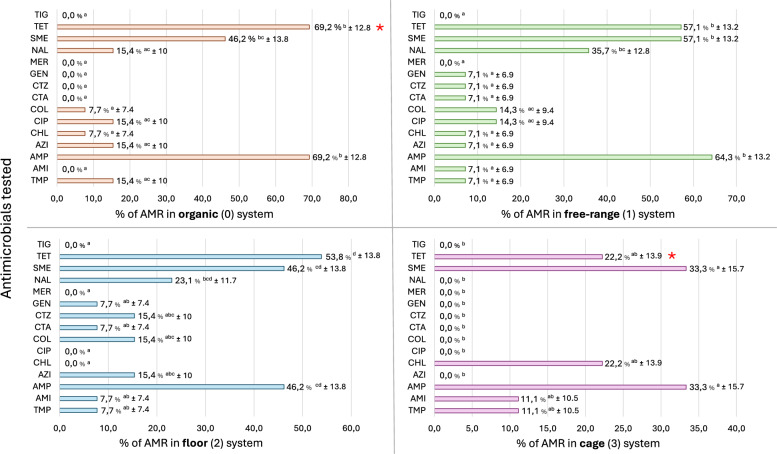
**Antimicrobial resistance of the** ***E. coli* isolates isolated from the different farms**. AMI, amikacin; AMP, ampicillin; AZI, azithromycin; CHL, chloramphenicol; CIP, ciprofloxacin; COL, colistin; CTA, cefotaxime; CTZ, ceftazidime; GEN, gentamicin; MER, meropenem; NAL, nalidixic acid, SME, sulfamethoxazole; TET, tetracycline; TIG, tigecycline;TMP, trimethoprim, ±  standard error, a–d: different superscripts in the same figure indicate statistically significant differences (*p* value < 0.05) between the antimicrobials studied, *: indicates statistically significant differences (*p* value < 0.05) for the same antimicrobial in different production systems.

Regardless of production system, the most common resistances were to AMP (55.1%), TET (53.1%) and SME (46.9%) (Figure 2). By contrast, no AMR was observed for MER or TIG, two last-resort antimicrobials in human medicine. Additionally, statistically significant differences were found to the quinolones group, as both free-range and floor *E. coli* isolates exhibited a higher prevalence of resistance than those from cage systems (*p* value < 0.05).

**Figure 3:**
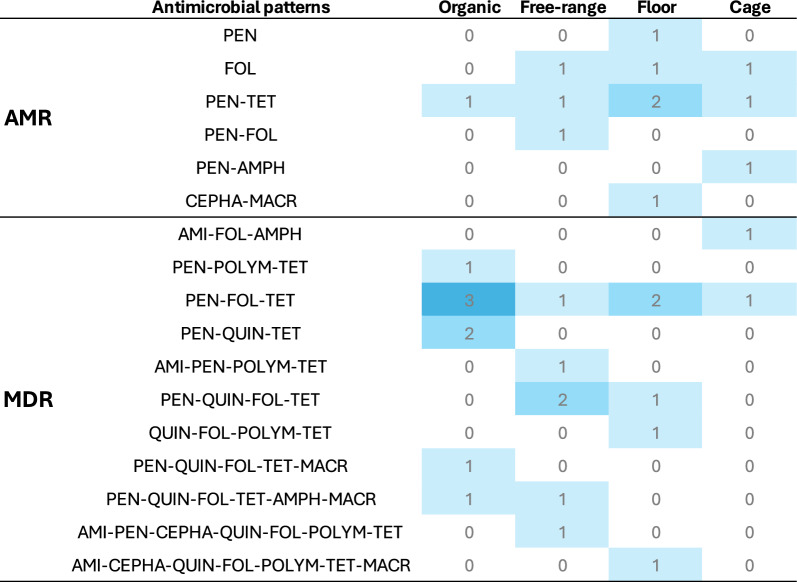
**Heatmap showing the distribution of antimicrobial resistance (AMR) and multidrug resistance (MDR) patterns among isolates from different production systems (organic, free-range, floor and cage)**. Numbers inside cells indicate the number of isolates presenting each resistance pattern. PEN, penicillins; FOL, folate inhibitor pathways; TET, tetracyclines; AMPH, amphenicols; CEPHA, cephalosporins; MACR, macrolides; POLYM, polymyxins; QUIN, quinolones. Resistance to three or more classes of antimicrobial is indicative of multidrug resistance.

Overall, 17 different AMR phenotypic patterns, grouped by antimicrobial class, were found (Figure 3). However, a clear pattern was observed for penicillins (PEN), folate inhibitor pathways (FOL) and tetracyclines (TET), which were present in almost all AMR patterns. The most prevalent one was PEN–FOL–TET (*n* = 7), followed by PEN–TET (*n* = 5). The only pattern observed in all the production systems were PEN–quinolones (QUIN)–FOL–TET (*n * = 4) and FOL alone (*n* = 3).

### *E. coli* molecular typing

**Figure 4:**
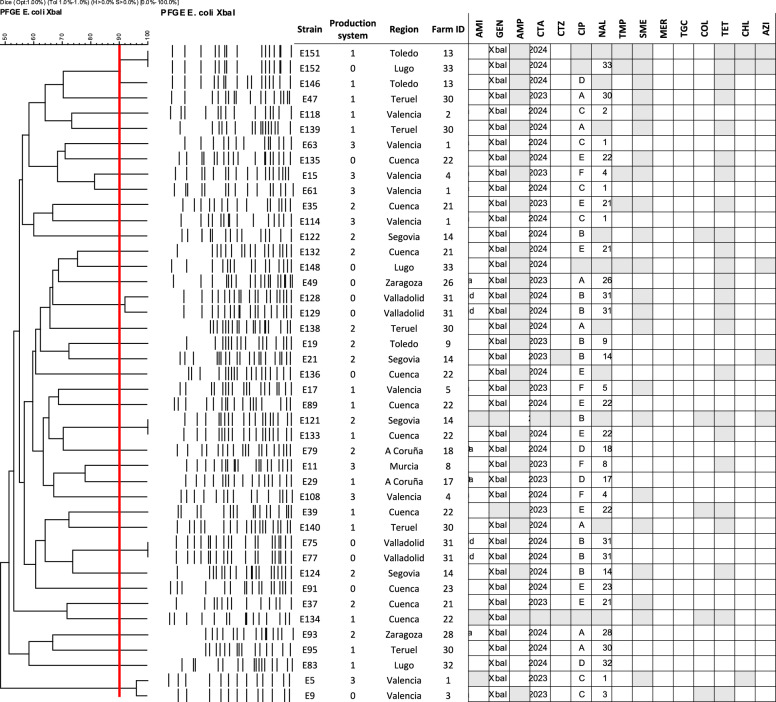
**PFGE dendrogram of XbaI profiles of** ***E. coli* and their antimicrobial resistances isolated from commercial eggs from different farms and commercial systems in Spain**. Production systems 0: organic, 1: free-range, 2: floor, 3: cage. Red line: indicates a similarity > 90% between isolates. Gray boxes: resistant. White boxes: susceptible. AMI: amikacin, GEN: gentamicin, AMP: ampicillin, CTA: cefotaxime, CTZ: ceftazidime, CIP: ciprofloxacin, NAL: nalidixic acid, TMP: trimethoprim, SME: sulfamethoxazole, MER: meropenem, TGC: tigecycline, COL: colistin, TET: tetracycline, CHL: chloramphenicol, AZI: azithromycin.

The PFGE analysis revealed a great genetic diversity with 37 different pulsotypes identified among the 49 isolates (Figure 4). Hence, most of the pulsotypes were singletons and only five pulsotypes included between two and four isolates (>90% similarity). Among the latter, two pulsotypes grouped isolates from the same farm (organic). The remaining three pulsotypes included isolates from different localities and different production systems: (1) organic and free range, (2) free-range and floor and (3) organic and cage. Also, different *E. coli* isolates (pulsotypes) were identified within the same farms. Moreover, isolates from the same pulsotype showed different AMR patterns, except those from farm ID31 where the two pulsotypes from this farm included two isolates each, with the same AMR profile (pan-susceptible and MDR, respectively).

## Discussion

Eggs and egg products have long been a key component of the human diet, playing a significant role in the food industry owing to their multifunctional properties and diverse forms of use and preparation [15]. The egg sector is undergoing a period of significant transformation and evolution, moving away from conventional cage-based systems towards alternative methods of hen housing. These new systems are characterised by the elimination of cages and the release of hens onto the ground, as well as allowing access to outdoor areas [7, 8]. Nevertheless, this engenders a considerable risk, as hens are in closer proximity to the environment and the wild animals that inhabit it. Consequently, they are more exposed to novel challenges in this sector, such as the transmission of certain diseases or the acquisition of AMR from nearby farms, streams or sewage.

Therefore, the present study aimed to compare the AMR in the different production systems in the egg sector. To this end, *E. coli* was used as a sentinel bacterium. The prevalence found in the eggshells (49%) and in the eggs’ content (0%) is consistent with the findings of other studies [15–17]. However, the United States Department of Agriculture, Food Safety and Inspection Services (USDA) and other authors, have described the presence of some microorganisms in the content of the egg. This is mainly attributed to the presence of some microorganisms, such as *E. coli*, within the hen’s ovary or oviduct, contaminating the inside of the egg during its formation [18].

When comparing the prevalence of *E. coli* among the different production systems, no differences were found, with similar prevalences among them [16, 17, 19, 20]. These results demonstrate the ubiquity of this bacterium and its significance in surveillance programmes, establishing it as a crucial ally that facilitates the monitoring of AMR, both at farm level and on a broader environmental scale. This study shows no statistically significant differences regarding the AMR observed across the different production systems, emphasizing the global burden AMR represents. Nevertheless, there were statistically significant differences (*p* value < 0.05) regarding the presence of MDR among systems, with *E. coli* isolated from eggs within organic systems exhibiting a higher MDR rate compared with the isolates from cage systems (61.5% versus 22.2%, respectively). It should be noted that in organic systems, hens are not treated with antimicrobials. In case of illness where treatment is necessary, any eggs produced cannot be sold as organic. Consequently, the primary hypothesis derived from the analysis of the present data is that exposure to the environment and other wild animals, or to their waste, enhances the probability of encountering MDR bacteria that colonise the gut microbiota which can then disseminate their AMR horizontally by means of mobile genetic elements. Therefore, bacteria isolated from eggs laid by organic hens demonstrate a higher degree of genetic variability in comparison with bacteria from eggs laid by hens raised in a controlled environment, where hens tend to exhibit a more homogeneous microbiota [16, 21].

Moreover, an analysis of the individual AMRs for each antimicrobial studied reveals discrepancies between the two systems, with higher prevalence of AMR for the same antimicrobial (e.g. TET) observed in organic systems. Nevertheless, regardless of the production system, the highest level of resistance was noted in AMP, TET and SME, as described by various authors [15, 21, 22]. Furthermore, the European Food Safety Authority (EFSA) has confirmed that the occurrence of AMR to these antimicrobials is a recurring pattern that has been observed throughout Europe, among different poultry species and their products [23, 24]. Moreover, other studies investigating AMR in laying hens in the Iberian Peninsula have reported the same patterns in both Spain [25] and Portugal [26], indicating that such resistance profiles are not uncommon on farms. The findings of the present study, which demonstrate a consistent pattern in the results of the antimicrobials studied across the European poultry sector, underscore the critical importance of environmental control in poultry farming. However, the solution does not only lie in restricting the use of antimicrobials on farms, as their presence in the environment is a significant source of resistance acquisition, with the consequent contamination of the final product intended for human consumption. It is therefore essential to implement rigorous biosecurity measures, encompassing not only the houses themselves, with stringent cleaning and disinfection protocols, but also the entire perimeter surrounding them [27].

On the other hand, restrictions and policies aimed at mitigating the emergence of AMR have resulted in a reduction in resistance levels to some highest priority critically important antimicrobials (HPCIAs), which are used to combat MDR infections in human medicine [24], as evidenced by the present study, where no AMR has been observed against colistin or meropenem.

In addition, PFGE analysis has revealed a great diversity of *E. coli* isolates. However, although some genetic similarities have been found between different production systems, different AMR patterns have been observed within the same pulsotype. These results emphasise the ubiquity of this bacterium and its ability to acquire AMR genes depending on the environment to which it is exposed [28].

In this context, where the sector is subject to constant change, it is imperative to adapt to consumer requirements, while respecting and improving animal welfare. At the same time, it is essential to recognise the challenges facing the sector with the emergence of alternative production systems. The findings of this study demonstrate that organic production systems exhibit a greater degree of MDR, probably owing to increased contact with the environment, wildlife and its waste, in comparison with cage systems. These results suggest that organic production systems are more susceptible to external environmental factors compared with conventional systems, which are characterised by restricted access to the exterior environment. However, further studies including a larger sample size are needed. In addition to egg samples, animals and farm environmental samples should also be analysed, as the farm environment plays a key role in the persistence and transmission of antimicrobial-resistant microorganisms. This approach would provide more representative information regarding the circulation of antimicrobial resistance among farms with different production systems.

## Supplementary information

**Additional file 1.** **Farms sampled per Spanish region, their corresponding identification number and production system, and the number of** ***Escherichia coli*** **isolates with their identification.**

## Data Availability

All data generated or analysed during this study are included in this published article [and its supplementary information files].
