# Supplementary material for: Epidemiology of antimicrobial resistance in commercial eggs across different production systems in Spain
Source: Vet Res. 2026 Jun 25;57:117. doi: 10.1186/s13567-026-01798-8 (PMC13307403; doi:10.1186/s13567-026-01798-8)
Supplement: Supplementary file 1 — Additional file 1. Farms sampled per Spanish region, their corresponding identification number and production system, and the number of Escherichia coli isolates with their identification. [file 13567_2026_1798_MOESM1_ESM.docx]

**Additional file 1.** Farms sampled per Spanish region, their corresponding identification number and production system, and the number of *Escherichia coli* isolates with their identification.

| **Region** | **Number of farms sampled per region** | **ID of each farm** | **Production system** | **ID of each *E. coli* isolate** |
| --- | --- | --- | --- | --- |
| A Coruña | 3 | 17 | 1 | E29 |
|  |  | 18 | 2 | E79, E81 |
|  |  | 19 | 1 | - |
| Cuenca | 4 | 21 | 2 | E35, E37, E132 |
|  |  | 22 | 1 | E39, E89, E133, E134, E135, E136 |
|  |  | 23 | 0 | E91 |
|  |  | 24 | 2 | - |
| Guadalajara | 1 | 35 | 2 | - |
| Lugo | 2 | 32 | 1 | E42 |
|  |  | 33 | 0 | E97, E152 |
| Madrid | 1 | 16 | 3 | - |
| Murcia | 1 | 8 | 2 | E11 |
| Pontevedra | 1 | 34 | 0 | - |
| Segovia | 1 | 14 | 0 | E21, E121, E122, E124 |
| Sevilla | 1 | 20 | 2 | - |
| Tarragona | 1 | 15 | 0 | - |
| Teruel | 1 | 30 | 1 | E47, E95, E138, E139, E140 |
| Toledo | 4 | 9 | 2 | E19 |
|  |  | 10 | 2 | E103 |
|  |  | 12 | 2 | - |
|  |  | 13 | 1 | E146, E151 |
| Valencia | 7 | 1 | 3 | E1, E61, E63, E114 |
|  |  | 2 | 3 | E118 |
|  |  | 3 | 0 | E9 |
|  |  | 4 | 3 | E15, E101, E108 |
|  |  | 5 | 1 | E17 |
|  |  | 6 | 1 | - |
|  |  | 7 | 0 | - |
| Valladolid | 1 | 31 | 0 | E75, E77, E127, E128, E129, E130 |
| Zaragoza | 5 | 25 | 2 | - |
|  |  | 26 | 0 | E49 |
|  |  | 27 | 1 | - |
|  |  | 28 | 2 | E93, E137 |
|  |  | 29 | 0 | - |
